# Pandemic influenza preparedness: an ethical framework to guide decision-making

**DOI:** 10.1186/1472-6939-7-12

**Published:** 2006-12-04

**Authors:** Alison K Thompson, Karen Faith, Jennifer L Gibson, Ross EG Upshur

**Affiliations:** 1 Centre for Research on Inner City Health, St. Michael's Hospital, 30 Bond Street, Toronto, Ontario, M5B 1W8, Canada; 2 Clinical Ethics Centre, Sunnybrook Health Sciences Centre, Toronto, Ontario M4N 3M5, Canada; 3 Joint Centre for Bioethics, University of Toronto, Toronto, Ontario, M5G 1L4, Canada; 4 Primary Care Research Unit, Sunnybrook Health Sciences Centre, and Joint Centre for Bioethics, Toronto, Ontario M5G 1L4, Canada

## Abstract

**Background:**

Planning for the next pandemic influenza outbreak is underway in hospitals across the world. The global SARS experience has taught us that ethical frameworks to guide decision-making may help to reduce collateral damage and increase trust and solidarity within and between health care organisations. Good pandemic planning requires reflection on values because science alone cannot tell us how to prepare for a public health crisis.

**Discussion:**

In this paper, we present an ethical framework for pandemic influenza planning. The ethical framework was developed with expertise from clinical, organisational and public health ethics and validated through a stakeholder engagement process. The ethical framework includes both substantive and procedural elements for ethical pandemic influenza planning. The incorporation of ethics into pandemic planning can be helped by senior hospital administrators sponsoring its use, by having stakeholders vet the framework, and by designing or identifying decision review processes. We discuss the merits and limits of an applied ethical framework for hospital decision-making, as well as the robustness of the framework.

**Summary:**

The need for reflection on the ethical issues raised by the spectre of a pandemic influenza outbreak is great. Our efforts to address the normative aspects of pandemic planning in hospitals have generated interest from other hospitals and from the governmental sector. The framework will require re-evaluation and refinement and we hope that this paper will generate feedback on how to make it even more robust.

## Background

As the world prepares for the emergence of a pandemic strain of influenza, trans-national, national and local organisations and agencies are designing plans to manage community outbreaks. In addition, the medical community is identifying scientific research priorities and needs related to the anticipated pandemic [1-5]. There is also a need to examine the ethical issues that arise from planning for a public health crisis of this magnitude. Who should get the limited supply of antivirals? Are health care workers duty-bound to care for the ill in a pandemic when they may have competing familial obligations? Who will be prioritized for scarce ventilated hospital beds? When should hospitals cancel elective surgeries or restrict hospital visitation? To date, the bioethics community has been slow to respond to public health issues in general [6,7], and pandemic influenza planning in particular [8,9]. In this paper we discuss the need for ethics in pandemic influenza planning and discuss the ethical framework we developed to guide pandemic planning in hospitals.

In the only article we could find that has an in-depth analysis of the ethics of pandemic planning, Kotalik offers an ethical analysis of the pandemic plans of three countries. His arguments are primarily about the ethics *of *pandemic planning efforts, as opposed to the ethics *in *pandemic planning. For example, he argues persuasively that it is problematic that all three countries' plans accept particular conditions of resource scarcity as planning assumptions [10]. While Kotalik has raised important issues about the ethics *of *pandemic planning in his article, our ethical framework focuses specifically on providing guidance to decision-makers about ethical issues *in *pandemic planning. This includes providing guidance on how to design an ethical process for decision-making, and providing guiding ethical values for the consideration of substantive issues.

The framework here proposed is an example of practical ethics that attempts to provide decision-makers with an introduction to and articulation of generally accepted ethical principles or values. The significance of this ethical framework is a) in the unique collaborative approach taken to its development that involved ethicists with different areas of expertise and a variety of health care stakeholders, and b) that it fills an important need in pandemic planning for an ethical framework to guide decision-making that has been unmet in most pandemic planning processes world wide.

### The importance of ethics in pandemic planning

One of the characteristics of a public health crisis is that health needs overwhelm available human and material resources. Difficult decisions must be made about how, where and to whom resources should be allocated. Medical science provides valuable information to help make these decisions. However, science alone is insufficient. Now consider that resource allocation decisions are just one kind of decision decision-makers face in preparing for, and getting through an influenza pandemic [9]. As a few scholars have begun to point out, pandemic planning needs to take ethical considerations seriously, and not allow the urgency of logistical and scientific needs to sideline a discussion of ethical considerations [10,11].

Kotalik argues that as "every discourse about health care has not only a scientific but also a moral dimension, [pandemic influenza] plans also presuppose certain ethical values, principles, norms, interests and preferences" [10]. It is important to make these presuppositions explicit, because, as the SARS experience in Toronto taught health care organisations, the costs of not addressing the ethical concerns are severe: loss of public trust, low hospital staff morale, confusion about roles and responsibilities, stigmatization of vulnerable communities, and misinformation [12-14]. Another key insight from SARS that we overlook at our peril was that in times of crisis, "where guidance is incomplete, consequences uncertain, and information constantly changing, where hour-by-hour decisions involve life and death, *fairness is more important, rather than less *[emphasis added]" [14]. As we shall argue, fairness considerations are both procedurally and substantively important: there is a need for fair decision-making processes, as well as equitable distributions of scarce human and material resources.

Take the example of triaging ventilated beds in an ICU. In theory, decision-makers rely on scientific evidence to determine how best to maximise benefit in the allocation of ventilated beds, but science cannot tell us whether or not the initial decision to maximise benefit is just. Because the notion of maximising benefit is derived from a reflection on values, ethical analysis is required to determine why a utilitarian approach to triage though maximisation of benefit is preferable to the assignment of ventilated beds on a different basis, for example that of greatest need. Even if the utilitarian maximisation of benefit is thought to be ethically sound, how to implement a system based on this criterion is not ethically straightforward, and requires ethical reflection about what counts as good stewardship, and about the moral obligation to demonstrate transparency, accountability, fairness and trustworthiness in the allocation of scarce resources.

The importance of ethics to pandemic planning is in the "the application of value judgements to science" [15], especially as they are embedded in planning assumptions, and within the practice of medicine itself. For example, while ethics might have little to contribute to understanding the mechanism of influenza virus transmission, it can make a significant contribution to debates such as what levels of harm the public are prepared to accept, how the burdens of negative outcomes should be distributed across the population and whether or not more resources should be invested in stockpiling antiviral medications.

The use of ethical frameworks to guide decision-making may help to mitigate some of the unintended and unavoidable collateral damage from an influenza pandemic. As Kotalik argues, the incorporation of ethics into pandemic plans can help to make them "instruments for building mutual trust and solidarity at such time that will likely present a major challenge to our societies" [10]. Using ethical frameworks to help guide decisions can offer greater assurance that the values instantiated within them, such as accountability, transparency and trust, will be carefully thought about in decision-making and when reviewing decisions with stakeholders.

## Discussion

### Development of the ethical framework

One of the key lessons from the Toronto SARS experience was that health care institutions and their staff could benefit from the development of ethical frameworks for decision-making [12]. The intention of this section is not to systematically derivate from normative theory the values and principles in the framework. This paper has a more narrow focus – it is an example of applied/practical ethics that attempts to introduce and articulate values that are already commonly accepted. It is not our intention to comprehensively defend the values in the framework, but rather to show from which areas of scholarship they were drawn, articulate their relevance to pandemic planning, and to demonstrate their discursive legitimacy through a process of stakeholder engagement and vetting. To our knowledge, no other pandemic planning process has attempted to a) develop an ethical framework to guide pandemic influenza planning and b) assess an ethical framework's robustness and resonance in the community of its intended users. Thus, the significance of the procedural elements of the development of the framework is not to be minimized, nor are the insights we have gleaned from implementing the framework in health care organisations and in a governmental setting.

Building on key lessons from SARS [12-14] and the "emergency ethics" literature and drawing on our expertise in clinical, organisational, and public health ethics, we identified key ethical processes and values that are relevant for health care organisations. These values were presented to and vetted by a variety of health care stakeholders. Thus, this framework is the product of an iterative and inclusive process.

#### Formation of a working group

In Ontario the need for guidance on the ethical issues pertaining to an influenza pandemic has been widely acknowledged. As word of our work on an ethical framework for Sunnybrook and Women's College Health Science Centre (S & W) became known, we were invited to join other hospitals' pandemic planning efforts. There was also broader sectoral interest in ethics, and we were invited to join the Ontario Ministry of Health and Long Term Care's (MOHLTC) efforts to design a pandemic plan.

Our working group was formed in response to the pandemic planning initiative that took place at S & W in early 2005. The hospital's Clinical Ethics Centre was invited to provide ethics support in this planning initiative. It soon became apparent that the scope of the issues went beyond the purview of clinical ethics to include organisational and public health ethics. Expertise in organisational and public health ethics was quickly procured through the University of Toronto Joint Centre for Bioethics which is a partnership between the University and sixteen affiliated healthcare organizations that includes S & W among its partners. S&W was subsequently de-amalgamated into Sunnybrook Health Sciences Centre and Women's College Hospital, thus the ethical framework is currently being implemented at Sunnybrook HSC.

As the framework took shape, we were invited to join the MOHLTC planning efforts. We began to work with the Vaccine and Antiviral working group at the MOHLTC, and we adapted our work to meet the related but distinct challenges facing government. While our work with the MOHLTC began with the Vaccine and Antiviral working group, the ethical framework we developed for the MOHLTC was eventually included in the *Ontario Health Pandemic Influenza Plan *[16] not as an annex to the section on vaccines and antivirals as we had originally anticipated, but as an ethical framework for the plan as a whole.

#### Review of clinical ethics and public health ethics literature

Expertise in clinical ethics was important to the development of this framework because of the knowledge, skills and experience clinical ethicists need to address dilemmas or challenges found in the daily clinical arena. An obvious challenge was how to integrate expertise in public health ethics into a framework designed to guide decision-making in clinical health care settings. A related challenge was to thoughtfully integrate generally accepted principles and values from clinical ethics with those in public health ethics. In order to meet this challenge, the authors turned not only to the respective ethics literature, but also to the SARS experiences of Toronto hospitals and health care providers. A review of the SARS literature, and that of public health ethics more generally, guided the integration of the public health and the clinical ethics perspectives [6,9,10,12-14,17-19]. The Toronto experience with SARS demonstrated that organisations faced unique ethical challenges when dealing with a public health crisis, and much of the ethics literature identified a need for greater forethought in how organisations can foster ethical decision-making in times of crisis [12-14]. We reasoned that the legitimacy of this framework would be enhanced by including insights from the analysis of a recent public health crisis like SARS.

#### Lessons from emergency ethics

Not surprisingly, the literature on clinical ethics has little to say about disaster preparedness and how to make decisions about such things as triage under extraordinary circumstances. The ethics literature on bioterrorism and battle-field triage informed our thinking and called our attention to important issues such as the duty to care, reciprocity, equity and good stewardship [20-25]. The importance of having ethically robust criteria and policies developed in advance of a pandemic influenza outbreak is underscored in this literature, for "critical decisions like these should not be made on an individual case-by-case basis" and "physicians should never be placed in a position of individually deciding to deny treatment to patients without the guidance of policy or protocol" [22]. Robust disaster preparedness requires practising preventive ethics.

#### Stakeholder vetting

The ethical framework was vetted through S & W's Pandemic Planning Committee, the Joint Centre for Bioethics' Clinical Ethics Group (comprised of the affiliated health care organizations' clinical ethicists), the MOHLTC Vaccine and Antiviral Working Group, and the MOHLTC pandemic planning committee. Through this process, we refined the framework and we are grateful to these groups for their valuable insights.

### The ethical framework

The ethical framework is intended to inform decision-making, not replace it. It is intended to encourage reflection on important values, discussion and review of ethical concerns arising from a public health crisis. It is intended also as a means to improve accountability for decision-making and may require revision as feedback and circumstances require.

The framework is divided into two distinct parts, and begins with the premise that planning decisions for a pandemic influenza outbreak ought to be 1) guided by ethical decision-making processes and 2) informed by ethical values. Ethical processes can help to improve accountability and it is hoped that, to the extent that it is possible for ethical processes to produce ethical outcomes, the substantive ethical quality of decisions will be enhanced. Recognising, however, that ethical processes do not guarantee ethical outcomes, we have identified ten key ethical values to guide decision-making that address the substantive ethical dimensions of decision-making in this context.

#### Ethical processes

In planning for and throughout a pandemic influenza crisis, difficult decisions will be made that are fraught with ethical challenges. Our framework around ethical processes is based upon the "accountability for reasonableness" model developed by Daniels & Sabin [26] and adapted by Gibson, Martin & Singer [27]. This model provides a useful means of identifying the key elements of ethical decision-making processes. An extensive literature has developed around Daniels' and Sabin's accountability for reasonableness framework. The Daniels and Sabin framework has broad applicability across institutional settings and priority setting situations [28-35]. Because the Daniels and Sabin framework applies deliberative theories of democratic justice to the specific problem of health care priority setting, and because it is unique in this regard, we felt it promoted the kind of deliberative approach to pandemic planning that this ethical framework is intended to support. Table 1 outlines the characteristics of an ethical decision-making process. Stakeholders will be more able to accept difficult decisions during a pandemic influenza crisis if the decision-making process has, and is perceived to have, ethical legitimacy.

**Table 1:** Ethical processes (Listed in alphabetical order). Adapted from Daniels, N. Accountability for reasonabless. BMJ 2000, 321:1300–1301.

**Value**	**Description**
**Accountability**	There should be mechanisms in place to ensure that ethical decision-making is sustained throughout the crisis.

**Inclusiveness**	Decisions should be made explicitly with stakeholder views in mind and there should be opportunities for stakeholders to be engaged in the decision-making process. For example, decision-making related to staff deployment should include the input of affected staff.

**Openness & Transparency**	Decisions should be publicly defensible. This means that the process by which decisions were made must be open to scrutiny and the basis upon which decisions are made should be publicly accessible to affected stakeholders. For example, there should be a communication plan developed in advance to ensure that information can be effectively disseminated to affected stakeholders and that stakeholders know where to go for needed information.

**Reasonableness**	Decisions should be based on reasons (i.e., evidence, principles, values) that stakeholders can agree are relevant to meeting health needs in a pandemic influenza crisis and they should be made by people who are credible and accountable. For example, decision-makers should provide a rationale for prioritising particular groups for anti-viral medication and for limiting access to elective surgeries and other services.

**Responsiveness**	There should be opportunities to revisit and revise decisions as new information emerges throughout the crisis as well as mechanisms to address disputes and complaints. For example, if elective surgeries are cancelled or postponed, there should a formal mechanism for stakeholders to voice any concerns they may have with the decision.

#### Ethical values

The second part of the framework identifies ten key ethical values that should inform the pandemic influenza planning process and decision-making during an outbreak. These values are intended to provide guidance, and it is important to consider that more than one value may be relevant to a situation. Indeed, the hallmark of a challenging ethical decision is that one or more value(s) are in tension and that there is no clear answer about which one to privilege in making the decision. When values are in tension with one another, the importance of having ethical decision-making processes is reinforced (see above.)

The values identified in our ethical framework were based initially on previous research findings on ethics and SARS at the University of Toronto Joint Centre for Bioethics (JCB). This work was funded by a Canadian Institutes of Health Research grant in 2004 through 2006 and has led to several key publications on the ethical dimensions of SARS [14,36-39]. In particular, Singer et. al., in their seminal British Medical Journal article begin to identify key ethical values that were of relevance during the SARS epidemic in Toronto. These values were then further articulated by our working group and adapted for the pandemic influenza planning context. Through a discursive process of stakeholder consultation with public health specialists, ministry officials, S & W's pandemic influenza committee, and the Clinical Ethics Group at the JCB, we augmented the values to include two new values (stewardship and trust [40,41]) and refined the definitions of each value in light of the anticipated demands of a pandemic influenza crisis compared to a hospital-based epidemic such as SARS. The substantive values identified and articulated in the framework are not intended to be an exhaustive set, and they may underdetermine how best to achieve the *overall *goals of pandemic planning, which generally include the minimization of morbidity, mortality, and societal disruption. Nevertheless, this is not to say that that a procedural engagement about the overall goals of a pandemic response would not benefit from using the ethical framework to guide and shape debate. A description of the values that should guide decision-making can be found in Table 2.

**Table 2:** Ethical values to guide decision-making (Listed in alphabetical order)

**Value**	**Description**	**Example**
**Duty to Provide Care**	The duty to provide care and to respond to suffering is inherent to all health care professionals' codes of ethics. In an influenza pandemic, demands on health care providers and the institutions in which they work will overwhelm resources. Health care providers will have to weigh demands from their professional role with other competing obligations to their own health, to family and friends. Health care workers will face significant challenges related to resource allocation, scope of practice, professional liability, and workplace conditions. *Decision makers should*: • Work collaboratively with stakeholders and professional colleges in advance of an influenza pandemic to establish practice guidelines • Work collaboratively to develop fair and accountable processes to resolve disputes • Provide supports to ease this moral burden of those with the duty to care • Develop means through which institutions will handle appeals or complaints, especially with regards to work exemptions, or the vaccination/prophylaxis of staff	Health care workers who are at increased risk because they are caring for patients with influenza must weigh familial obligations, and obligations to self with their professional duty to care. In addition, they may also have to comply with vaccination or antiviral regimens for prophylaxis which may conflict with their individual liberty.

**Equity**	The principle of equity holds that, all things being equal, all patients have an equal claim to receive needed health care. During influenza pandemic, however, tough decisions will need to be made about which health services to maintain and which to defer because of extraordinary circumstances. Measures taken to contain the spread of a deadly disease will inevitably cause considerable collateral damage. In an influenza pandemic, this will extend beyond the cessation of elective surgeries and may limit the provision of emergent or necessary services. *Decision-makers must strive to*: • Preserve as much equity as possible between the interests of patients [afflicted with the influenza] and those who need urgent treatment for other diseases • Ensure procedural fairness in decision-making	In allocating scarce resources, the value of equity could guide in developing fair criteria for allocation while consideration is given also to compensation for those who will not meet inclusion criteria yet are entitled to receive care.

**Individual Liberty**	Individual liberty is a value enshrined in health care practice under the principle of respect for autonomy. Under usual circumstances, health care providers balance respect for individual autonomy with a duty to protect individual patients from harm. In a public health crisis, however, restrictions to individual liberty may be necessary to protect the public from serious harm. Patients, staff, and members of the public may all be affected by such restrictions. *Restrictions to individual liberty should*: • Be proportional to the risk of public harm • Be necessary and relevant to protecting the public good • Employ the least restrictive means necessary to achieve public health goals • Be applied without discrimination	Social distancing strategies that employ visitor restrictions in hospitals must be necessary for the protection of the public and must be proportionate to the threat being allayed.

**Privacy**	Individuals have a right to privacy in health care. In a public health crisis, it may be necessary to override this right to protect the public from serious harm. A proportionate response to the need for private information requires that it be released only if there are no less intrusive means to protect public health. *Decision makers should*: • Disclose only private information that is relevant to achieve legitimate and necessary public health goals • Release private information only if there are no less intrusive means to protect public health • Determine whether the good that is intended is significant enough to justify the potential harm that can come from suspending privacy rights, (e.g. the harm from stigmatization of individuals or particular communities) • Provide public education to correct misconceptions about disease transmission and to offset misattribution of blame to particular communities	The need to conduct contact tracing of possibly infected people might require that particular groups or even individuals are identified publicly. The need to do so must be weighed against the potential harm of exposing communities and individuals to stigmatization.

**Proportionality**	Proportionality requires that restrictions to individual liberty and measures taken to protect the public from harm should not exceed what is necessary to address the actual level of risk to, or critical need of, the community. *Decision makers should*: • Use least restrictive or coercive measures in limiting or restricting liberties or entitlements • Use more coercive measures only in circumstances where less restrictive measures have failed to achieve appropriate public health ends.	The decision to close an emergency room must consider if the potential harm in keeping the emergency room open is significant enough to warrant its closure.

**Protection of the Public from Harm**	A foundational principle of public health ethics is the obligation to protect the public from serious harm. This principle requires that citizens comply with imposed restrictions in order to ensure public wellbeing or safety. To protect the public from harm, hospitals may be required to restrict public access to service areas (e.g. restricted visiting hours), to limit availability of some services (e.g. elective surgeries), or to impose infectious control practices (e.g. masks or quarantine). *When making decisions designed to protect the public from harm, decision makers should*: • Weigh the medical and moral imperative for compliance • Ensure stakeholders are made aware of the medical and moral reasons for public health measures • Ensure stakeholders are aware of the benefits of compliance & the consequences of non-compliance • Establish mechanisms to review these decisions as the public health situation changes and to address stakeholders concerns or complaints	When making the decision to quarantine individuals, protection of the public from harm must be weighed against individual liberty. Note that while the ethical value of individual liberty is often in tension with the protection of the public from harm, it is also in individuals' interests to minimize harm to others.

**Reciprocity**	Reciprocity requires that society supports those who face a disproportionate burden in protecting the public good and takes steps to minimise their impact as far as possible. In an influenza pandemic, measures to protect the public good are likely to impose a disproportionate burden on health care workers, patients, and their families. Health care workers may face expanded duties, increased workplace risks, physical and emotional stress, isolation from peers and family, and in some cases, infection leading to hospitalization or even death. Similarly, quarantined individuals or families of ill patients may experience significant social, economic, and emotional burdens. *Decision-makers and institutions are responsible for*: • Easing the burdens of health care workers, patients, and patient's families in their hospitals and in coordination with other health care organizations • Ensuring the safety of their workers, especially when redeploying staff in areas beyond the usual scope of practice	The provision of antiviral medication and/or vaccination to hospital staff for prophylaxis is one way hospitals can ensure the safety of their workers who may be exposed to greater than usual risks in discharging their duty to care.

**Solidarity**	SARS heightened the global awareness of the interdependence of health systems and the need for solidarity across systemic and institutional boundaries in stemming a serious contagious disease. An influenza pandemic will not only require global solidarity, it will require a vision of solidarity within and between health care institutions. *Solidarity requires*: • Good, open and honest communication • Open collaboration, in a spirit of common purpose, within and between health care institutions • Sharing public health information • Coordinating health care delivery, transfer of patients, and deployment of human and material resources	Territoriality between hospital departments and between health care institutions needs to be overcome with good communication and sense of common purpose in order to provide equitable care across jurisdictions

**Stewardship**	In our society, both institutions and individuals will be entrusted with governance over scarce resources, such as vaccines, antivirals, ventilators, hospital beds and even health care workers. During a pandemic influenza outbreak, difficult decisions about how to allocate material and human resources will have to be made, and there will be collateral damage as a result of these allocation decisions. Those entrusted with governance roles should be guided by the notion of stewardship. Inherent in stewardship are the notions of trust, ethical behaviour, and good decision-making. *Decision makers have a responsibility to*: • Avoid and/or reduce collateral damage that may result from resource allocation decisions • Maximize benefits when allocating resources • Protect and develop resources where possible • Consider good outcomes (i.e. benefits to the public good) and equity (i.e., fair distribution of benefits & burdens)	A hospital's decision to stock-pile antiviral medication must consider whether this is an effective way of protecting staff from infection, where the money for stockpiling will come from, and whether that money could be put to better use elsewhere.

**Trust**	Trust is an essential component in the relationships between clinician and patient, between staff and the organization, between the public and health care providers, and between organizations within a health system. In a public health crisis, stakeholders may perceive public health measures as a betrayal of trust (e.g. when access to needed care is denied) or as abandonment at a time of greatest need. Decision-makers will be confronted with the challenge of maintaining stakeholders' trust while at the same time stemming an influenza pandemic through various control measures. It takes time to build trust. *Decision-makers should*: • Take steps to build trust with stakeholders before the crisis hits not while it is in full swing • Ensure decision making processes are ethical and transparent to those affected stakeholders	Early engagement with stakeholders may go some distance to justify stakeholder confidence in decision-makers' trustworthiness. In part, the value of trust is respected and promoted by following the ethical *processes *outlined above.

Included in the framework are "hot button" ethical issues that we identified through our work with Toronto hospitals and the MOHLTC. These issues were as follows:

a) Targeting and prioritizing populations for vaccines and antivirals

b) Intensive Care Unit and hospital bed assignment

c) Duty to care

d) Human resources allocation and staffing

e) Visiting restrictions

f) Communications and how reviews of decisions will be handled

These "hot button" issues are not intended to be exhaustive, but rather they serve to illustrate how the values in the ethical framework can be used to identify key ethical aspects of decision-making.

Let us take the issue of targeting and prioritizing populations for vaccine and antivirals to illustrate how the values in the ethical framework can help guide decision-making. The values of solidarity and protecting the public from harm would require that priorities be set to maximize the capacity to help society ensure that the ill are cared for during a pandemic. Furthermore proportionality would require that decision-makers consider who within the community are most vulnerable to the contagion as well as who are most likely to benefit from immunization. A well-informed public conversant with the values in the ethical framework and aware of the expertise that informed the ranking of priorities for immunisation would be consistent with value of trust and the principle of transparency.

Lastly, while knowing how to use the framework to inform decision-making is vital, there is more to ensuring that the framework will be used or useful.

### Lessons for implementing an ethical framework

We have identified three necessary, if not exhaustive elements to the successful integration of ethics into hospital pandemic planning processes. These elements are 1) sponsorship of the ethical framework by senior hospital administration; 2) vetting of the framework by key stakeholders and; 3) decision review processes.

#### Sponsorship by senior administrators

Whether or not an ethical framework is used to inform decision-making in a health care institution depends to a large extent on people in senior positions of an organisation seeing its relevance to the decision-making process. In part, this is dependant on how robust the framework is, but it also requires the willingness to frame (at least some) pandemic planning issues as normative in nature.

Some may argue that the values in the framework are too stringent or impractical to implement under crisis conditions, especially those found in the Ethical Processes part of the framework (see Table 1). Certainly, crisis conditions may place constraints on the extent to which each principle can be acted upon. However, efforts should be made to put them into action to the fullest extent possible under the circumstances and in our experience this is only possible with the support of senior administrators.

The senior administration at S & W (many of whom were part of the Pandemic Planning Committee) had previous experience with the accountability for reasonableness framework for decision-making, and thus their pandemic influenza planning committee was already familiar with the Ethical Processes part of the framework, and they were receptive to the idea of being guided by an ethical framework. Senior administrators may also have been receptive to the ethical framework because, as they learned from SARS, organisations that did not have decision-making processes that honoured the values for ethical process during SARS have been dealing with a legacy of collateral damage to staff and patients in the form of distrust and low morale [12]. For these reasons, the senior administrators at S & W played an important role in vetting the ethical framework. Ensuring that institutional "sponsors" are in favour of adopting an ethical framework is important for gaining widespread support for using an ethical framework in decision-making, and for ensuring that the ethical framework does not become something that looks good but remains unused.

#### Vetting of the ethical framework by key stakeholders

In order to obtain support for, or "buy in" to an ethical framework, it is important that key stakeholders in an institution vet the framework. This requires careful consideration of who the key stakeholders are in an institution. Not only should this include those with responsibility for decision-making, but also those who will be affected by decisions taken. For the vetting process is not just intended to create "buy in" but also to decrease the likelihood that interests and issues that are (morally) relevant to pandemic planning will be neglected or overlooked, thereby enhancing the moral legitimacy of the values in the framework. In addition, a process of stakeholder vetting increases the likelihood that the values instantiated in the framework resonate with the stakeholder community.

It has been our experience that the values in the framework did resonate with the pandemic planners with whom we have shared this ethical framework. The primarily pragmatic justification for the selection of the values in the framework means that the framework is provisional so it ought to be subject to revision in light of compelling argument, empirical evidence and further stakeholder feedback. It is important to note, however, that the iterative and inclusive process through which the values in the framework were deliberated amongst the various stakeholder groups lends them a form of discursive ethical legitimacy and helps to justify their inclusion in the ethical framework. We intend that the framework invite further dialogue about its legitimacy and its adequacy. We will return to this issue in the final section of this paper.

Ideally, the vetting process would include people who can represent the interests of patients, families and volunteers who are part of the hospital's constituency. Although patient relations, human resources and occupational health representatives from S & W provided guidance and feedback in the development of the framework, direct input from patients and family representatives was not obtained. One limitation of our framework is that is has yet to be vetted by these important stakeholders.

The importance of solidarity to the management of a public health crisis would also suggest that the public and other health care organisations be considered stakeholders in hospital pandemic planning. While it may not be pragmatic for hospitals to undertake broad public consultation and vetting processes for their pandemic plans in general, and their ethical frameworks in particular, solidarity and equity suggest that these broader stakeholder interests are relevant to pandemic planning. Consequently, opportunities for broader ethical dialogue about pandemic planning need to be encouraged.

#### Decision review processes

In order to ensure that the support of key stakeholders is maintained through an outbreak, there need to be effective communication mechanisms in place. An important aspect of responsive decision-making processes is ensuring that there are formal opportunities to revisit and revise decisions as new information emerges. As part of our ethical framework, we formulated a template for decision review processes, (adapted from, Gibson, JL: *Formal decision review process template*. Unpublished; 2003) that aids organisations in identifying existing and establishing new mechanisms that can be used for the formal reviews of decisions. We believe decision review mechanisms are an essential part of ethical decision-making in a public health crisis, and are one way to put the values in the ethical framework in to action.

Formal mechanisms for reviewing decisions are needed in order to capture feedback from stakeholders on key decisions, and to resolve disputes and challenges. These processes are important for ensuring that decisions are the best possible under the circumstances given changing information and for engaging stakeholders constructively around the difficult decisions that must be made. Given the unpredictable nature of public health emergencies and the difficulty this poses for those in charge of planning and decision-making, it is reasonable to assume that decisions will be revised throughout the pandemic influenza crisis. Disputes or challenges may arise from the restrictions or requirements imposed on staff, patients and families during a pandemic influenza outbreak. Thus, decision review processes are essential. Again, while some may argue that this is too stringent a measure for a time of crisis, we argue that reviews of decisions will be taking place regardless (most likely in an *ad hoc *manner), and that to formalize this process is to increase its fairness and moral legitimacy. Indeed, there may be existing mechanisms which can handle these kinds of reviews.

### Scope of the ethical framework

It is important to distinguish between different types of ethical analyses in order to explain the approach that was taken to the development of the ethical framework discussed herein. Callahan and Jennings draw a useful distinction between *applied *ethics and *critical *ethics [7]. Our ethical framework is an example of applied ethics because the framework identifies and relies on "general principles that can be applied to real-world examples of professional conduct or decision-making"[7] and because it is "designed to give professionals guidance and to give clients and the general public standards to use in assessing professional conduct" [42]. While there is certainly a need for critical ethical analysis that pays attention to problems that are the "result of institutional arrangements and prevailing structures of cultural attitudes and social power" [7], one would not expect a ethical framework designed to guide clinical decision-making to explicitly address these kinds of issues.

This is not to say that this ethical framework *cannot *address the kinds of issues that a critical ethical analysis might address. For example, the framework promotes values and processes that seek to redress the power disparities within institutions. The section of the framework that deals with ethical processes in particular is a challenge to how institutional decisions are typically made. For example, the value of "inclusiveness" as a process principle is essential for redressing power differences amongst key stakeholders [27]. Thus, while the ethical framework is the product of applied ethical analysis, and should be evaluated in light of this, one of its strengths is that it can also redress what Callahan and Jennings would characterize as "critical" ethics problem of power disparities within institutions.

## Conclusion

### Cultural limitations and future directions

Within pluralistic societies, there are many different ethical perspectives that exist simultaneously on issues about global, public and individual health. An ethical framework to guide decision-making is robust to the extent that it reflects the values and beliefs of the decision-makers who refer to it and the values and beliefs of those affected by the decisions being taken. Our framework relied heavily on the Toronto experience with SARS to surface and examine the ethical values that are important for a public health crisis. An influenza pandemic is likely to present us with particular ethical challenges that are different from SARS due to the predicted severity of the contagion and its spread to the community. It would therefore be important not to uncritically adopt such a framework but rather to use it as a basis for continued reflection and re-evaluation to ensure its relevance and responsiveness during the unfolding health crisis. It is also important to consider the extent to which an ethical framework is reflective of the community in which it is to be used. Lessons from SARS as it was experienced in China would likely surface some different ethical values, or emphasise different aspects of our framework. As Callahan and Jennings have argued:

We submit that a rich discourse on ethics and public health cannot be advanced without relating it to the background values of the general society, and the particular communities, in which it will be carried out.[7]

Indeed, as previously maintained, there are many issues related to pandemic influenza planning – particularly those raised by a *critical *ethical analysis – that require broad public debate. While these kinds of issues require public debate that takes place at the societal level, ethical pandemic planning requires that organisations and agencies foster internal dialogue about the values instantiated in an ethical framework. For it is imperative that the values outlined in a framework resonate with the members of an organisation, and the community it serves. The procedural aspects of the framework provide a means to ensuring that the values of the community are reflected in decision-making through the procedural principles of inclusiveness and responsiveness.

It is important, too, to recognise that values are not static, and that circumstances will evolve rapidly during a pandemic influenza outbreak. Ethical frameworks will also require re-evaluation and revision. The challenge will be to continue to recognise the importance of moral reflection under circumstances that are not conducive to it and to encourage a process of re-evaluation that strives to assess whether resulting decisions are consistent with those values the framework is intended to promote. For this reason, it is imperative to start the ethical dialogue in advance, and to find ways to encourage consideration of ethical issues at all stages of decision-making. We hope that this paper will go some way towards advancing this objective, and that this paper stimulates discussion of the ethical issues and values that pervade pandemic planning.

We believe that this framework is unique in its blending of clinical, public health, and organizational ethics. One of its strengths is that it draws on lessons from the recent public health crisis of SARS in Toronto, and it is to some extent empirically grounded. Another strength is that it is the product of an inclusive process of development that included stakeholder vetting. It is also unique in its attempt to provide guidance to decision-makers facing a public health crisis. We hope that the framework's acceptance by hospitals and the provincial government in Ontario signals a change in the way that decisions are taken by institutions that are charged with making decisions that have life and death consequences for the public.

## Summary

• Good pandemic planning requires reflection on values because scientific information alone cannot drive decision-making.

• The development of an ethical framework for hospital pandemic planning calls for expertise in clinical, organisational and public health ethics.

• Stakeholder engagement is essential for the ethical framework to be relevant and legitimate.

• The ethical framework contains procedural and substantive ethical values to guide decision-making.

• Three key elements of integration of ethics in to pandemic planning are 1) sponsorship from senior hospital administration; 2) vetting by stakeholders and; 3) decision review processes.

• An ethical framework is robust to the extent that pandemic influenza planning decisions are seen to be ethically legitimate by those affected by them.

• In order to increase the robustness of pandemic planning in general, timely public debate about the ethical issues is essential.

## Competing interests

The author(s) declare that they have no competing interests.

## Authors' contributions

AT, KF, JG and RU contributed equally to the development of the ethical framework. AT drafted this manuscript and KF, JC and RU contributed equally to the revision of the manuscript. All authors read and approved the final manuscript.

## Pre-publication history

The pre-publication history for this paper can be accessed here:



## References

[B1] Stohr K (2005). Avian influenza and pandemics: research needs and opportunities. N Engl J Med.

[B2] Osterholm MT (2005). Preparing for the next pandemic. N Engl J Med.

[B3] Tam T, Sciberras J, Mullington B, King A (2005). Fortune favours the prepared mind: a national perspective on pandemic preparedness. Can J Public Health.

[B4] Reichert TA (2005). Preparing for the next influenza pandemic. The Pediatric Infectious Disease Journal.

[B5] Wong S, Yuen K (2006). Avian influenza virus infections in humans. Chest.

[B6] Beauchamp E, Steinbock B (1999). New Ethics for the Public's Health.

[B7] Callahan D, Jennings B (2002). Ethics and public health: forging a strong relationship. American Journal of Public Health.

[B8] Kotalik J (2005). Preparing for an influenza pandemic: ethical issues. Bioethics.

[B9] Zoloth L, Zoloth S (2006). Don't be chicken: bioethics and avian flu. The American Journal of Bioethics.

[B10] Kotalik J (2003). Addressing issues and questions relating to pandemic influenza planning: final report and recommendations.

[B11] Tracy SC, Upshur R, Daar A (2005). Avian influenza and pandemics. N Engl J Med.

[B12] Berstein M, Hawryluck L (2003). Challenging beliefs and ethical concepts: the collateral damage of SARS. Critical Care.

[B13] Singer P, Benatar S, Berstein M, Daar A, Dickens B, MacRae S, Upshur R, Wright L, Zlotnick Shaul R (2003). Ethics and SARS: lessons from Toronto. BMJ.

[B14] Bell J, Hyland S, DePelligrin T, Upshur R, Berstein M, Martin D (2004). SARS and hosptial priority setting: a qualitative case study and evaluation. BMC Health Services Research.

[B15] Perhac R (1998). Comparative risk assessment: where does the public fit in?. Science, Technology and Human Values.

[B16] MOHLTC (2005). Ontario Health Pandemic Influenza Plan.

[B17] Upshur R (2002). Principles for the justification of public health intervention. Can J Public Health.

[B18] Gostin LO (2001). Public health, ethics, and human rights: a tribute to the late Johnathan Mann. J Law Med Ethics.

[B19] O'Neill O (2002). Public Health or Clinical Ethics: Thinking beyond Borders. Ethics & International Affairs.

[B20] Wynia MK, Gostin LO (2004). Ethical challenges in preparing for bioterrorism: barriers within the health care system. Am J Public Health.

[B21] Iserson K, Pesik N (2003). Ethical resources distribution after biological, chemical or radiological terrorism. Cambridge Quarterly of Healthcare Ethics.

[B22] Pesik N, Keim M, Iserson K (2001). Terrorism and the ethics of emergency medical care. Annals of Emergency Medicine.

[B23] Veatch R (2005). Disaster prepardeness and triage: justice and the common good. Mt Sinai J Med.

[B24] Kipnis K (2003). Overwhelming casualties: medical ethics in a time of terror. Accountability in Research.

[B25] Marer S, Sutjita M, Rajagopalan S (2004). Bioterrorism, bioethics and the emergency physician. Topics in Emergency Medicine.

[B26] Daniels N (2000). Accountability for reasonableness. BMJ.

[B27] Gibson J, Martin D, Singer P (2005). Priority setting in hospitals: fairness, inclusiveness and the problem of institutional power differences. Social Science and Medicine.

[B28] Ham C (1999). Tragic choices in health care: lessons from the Child B case.. British Medical Journal.

[B29] Norheim OF (2000). Procedures for priority setting and mechanisms of appeal in the Norwegian health care system: ; Amsterdam..

[B30] Daniels N, Sabin J (2002). Setting limits fairly: can we learn to share scarce resources?.

[B31] Martin DK, Giacomini M, Singer P (2002). Fairness, accountability for reasonableness and the views of priority setting decision-makers. Health Policy.

[B32] Martin DK, Shulman K, Santiago-Sorrell P, Singer P (2003). Priority setting and hospital strategic planning: a qualitative case study. Journal of Health Services Research and Policy.

[B33] Gibson J, Martin DK, Singer P (2004). Setting priorities in health care organisations: criteria, processes, and parameters of success.. BMC Health Services Research.

[B34] Gibson J, Martin DK, Singer P (2005). Priority setting in hospitals: fairness, inclusiveness, and the problem of institutional power differences. Social Science and Medecine.

[B35] Mitton C, Donaldson C (2004). Health care priority setting: principles, practice and challenges. Cost Eff Resour Alloc.

[B36] Nickell LA, Crighton EJ, Tracy CS, Al-Enazy H, Bolaji Y, Hussain A, Makhlouf S, Upshur R, Hanjrah S (2004). Psychosocial effects of SARS on hospital staff: survey of a large tertiary care institution. Canadian Medical Association Journal.

[B37] Ruderman C, Tracy CS, Bensimon CM, Bernstein M, Hawryluck L, Zlotnick Shaul R, Upshur REG (2006). On pandemics and the duty to care: whose duty? who cares?. BMC Medical Ethics.

[B38] Singer P, Benatar S, Bernstein M, Daar A, Dickens B, MacRae S, Upshur R, Wright L, Zlotnick ShaulR (2003). Ethics and SARS: lessons from Toronto. British Medical Journal.

[B39] Wilson K, McDougall C, Upshur R, Joint Centre for Bioethics SARS Global Health Ethics Research Group (2006). The new International Health Regulations and the federalism dilemma. PLoS Med.

[B40] Saltman RB, Feroussier-Davis O (2000). The concept of stewardship in health policy. Bulletin of the World Health Organization.

[B41] Goold SD (2001). Trust and the ethics of health care institutions. The Hastings Centre Report.

[B42] Jennings B (2003). Frameworks for ethics in public health. Acta Bioethica.

